# Impact of Maternal Dental Anxiety on Oral Health-Related Quality of Life of Three-to-Six-Year-Old Children: A Cross-Sectional Study

**DOI:** 10.7759/cureus.107335

**Published:** 2026-04-19

**Authors:** Sayali Pate, Mansi Mangela, Amar N Katre

**Affiliations:** 1 Pediatric and Preventive Dentistry, Dr. G.D. Pol Foundation Y.M.T. Dental College and Hospital, Navi Mumbai, IND

**Keywords:** children, corah’s dental anxiety scale, ecohis, maternal dental anxiety, oral health related quality of life

## Abstract

Purpose: One of the common reasons for avoiding dental treatment in children is dental anxiety. Maternal dental anxiety is considered one of the determinants of children’s dental anxiety. This may have an impact on the oral health-related quality of life (OHRQoL) of children. This study aimed to assess the association of maternal dental anxiety with the OHRQoL of three-to six-year-old children.

Methods: Six hundred and fifty mother-child pairs participated in this cross-sectional study. Maternal dental anxiety was assessed using Corah’s Dental Anxiety Scale (CDAS). Parent-reported OHRQoL was assessed using the Early Childhood Oral Health Impact Scale (ECOHIS). Both scores were assessed as continuous variables, and a mean with SD was calculated. Maternal education and socioeconomic status (SES) were assessed using the modified Kuppuswamy scale. Pearson’s correlation coefficient was used to assess the association between CDAS scores and ECOHIS scores of children. ANOVA was used for intergroup comparison of CDAS scores and ECOHIS scores based on children’s age, sex, maternal age, maternal education, and SES.

Results: A significant positive correlation was observed between the scores of CDAS and ECOHIS in the child domain (r=0.476; p < 0.001), family domain (r=0.38; p < 0.001), and total (r=0.422; p < 0.001). Mean ECOHIS scores in children with high maternal dental anxiety were higher than those with low maternal dental anxiety (t=10.420; p < 0.0001), suggestive of a poor OHRQoL.

Conclusions: Maternal dental anxiety had a negative impact on the OHRQoL of children.

## Introduction

Anxiety is a state of apprehension, tension, or uneasiness arising from the anticipation of danger, often from an unknown source. It involves helplessness, lack of control, and the readiness to counter threats [1]. ‘Dental anxiety’ refers to stress, specific to dental situations, occurring before visits or treatment. Despite advancements in dentistry, it ranks among the most commonly feared situations [2], particularly in children, posing a public health challenge [3] and hindering the successful treatment of children [4]. It often leads to treatment avoidance, which worsens oral health, necessitates invasive and costly procedures, and reinforces anxiety. Studies show that 68% of highly anxious children have over five carious lesions by age five [5].

Poor oral health impacts daily functions, speech, and oral health-related quality of life (OHRQoL). Several oral diseases, including dental caries, periodontal disease, and malocclusion, can impact the OHRQoL. Children with untreated dental caries often suffer from reduced OHRQoL [6]. Dental anxiety may result in avoidance of dental treatment and thus poor OHRQoL [6]. The etiology of dental anxiety in children is multifactorial, with a combination of exogenous contributory factors [7] and endogenous factors, including personality traits [1]. The most relevant of these contributing factors is parental anxiety. Children are as susceptible to anxiety as adults, and their anxiety is derived from peer communication of reported bad experiences or even threats made by parents [8]. Maternal dental anxiety has been reported to be associated with child anxiety and functions as an indicator of the child’s oral health behaviors and dental service use [9]. A strong association between children’s dental anxiety and parental, particularly maternal anxiety, has also been observed [10,11].

However, limited research exists on the relation between maternal dental anxiety and children’s OHRQoL, highlighting the need for this study. The primary objective of the study was to assess the association between maternal dental anxiety and OHRQoL in three-to six-year-old children. The secondary objectives were to assess the association of maternal age, maternal education, and socioeconomic status (SES) with maternal dental anxiety and OHRQoL in three-to-six-year-old children.

## Materials and methods

This cross-sectional study was conducted, as per the Strengthening the Reporting of Observational Studies in Epidemiology (STROBE) checklist [12], on three-to-six-year-old children reporting to the Department of Pediatric and Preventive Dentistry at the Dr. G.D. Pol Foundation Y.M.T. Dental College and Hospital, Navi Mumbai, India, from November 2022 to November 2023, either for treatment or for a routine dental check-up; also participating were children (in lower kindergarten, upper kindergarten, and first standard classes) from a private school in the vicinity of the dental college, along with their mothers. Healthy children between three and six years of age and their mothers were included. Children who were physically or mentally challenged or medically compromised, whose parents refused to give consent, and mothers whose cognitive ability interfered with data collection were excluded.

Information sheets were given to the parents stating the complete details of the study and their rights related to participation. Prior to commencing study, written consent was obtained. All the documents were available in English and the local vernacular language. Institutional Ethics Committee, Dr. G.D. Pol Foundation Y.M.T. Dental College and Hospital issued approval YMTDC&H/IEC/2022/131.

Maternal dental anxiety, the main exposure variable, was assessed using Corah’s Dental Anxiety Scale (CDAS) [13]. The scale included four questions focused on the individual’s subjective reactions to dental visits, waiting at the dentist’s office, and anticipated drilling and scaling. Each question had five answer choices, which were assigned a score from 1 to 5. Any query of mothers regarding the scale was answered by the primary investigator. The scores ranged from 4 to 20.

The Early Childhood Oral Health Impact Scale (ECOHIS) was used to evaluate the OHRQoL of children [14]; the questionnaire was filled out by the mothers. The ECOHIS consisted of 13 items organized into two sections: the Child Impact Section (CIS) and the Family Impact Section (FIS). The CIS included four subscales: child symptoms, child function, child psychology, child self-image, and social interaction [14]. The FIS included two subscales: parental distress and family function. The questionnaire used a five-point rating scale to indicate the frequency of events in a child’s life: 0=never; 1=hardly ever; 2=occasionally; 3=often; 4=very often; 5=do not know. ECOHIS scores ranged from 0 to 36 in the CIS category and 0 to 16 in the FIS category [14]. Higher scores suggested a greater impact on oral health and a poorer OHRQoL [14].

The scores of CDAS and ECOHIS were recorded as continuous variables, and a mean with a standard deviation was calculated. A cutoff score of 11 was used to categorize maternal dental anxiety into high and low [14].

The mother’s education and the SES of the family were recorded and categorized using the Modified Kuppuswamy Scale [15]. The Maternal Education Subscale of the Modified Kuppuswamy scale scores, Modified Kuppuswamy Scale scores, and sex were depicted as frequencies with percentages.

The sample size was estimated based on the reported data for the association of maternal dental anxiety with OHRQoL (RR=1.45) [10]. Assuming the lower limit of acceptable association to be 1.35, a sample size of 603 subjects achieved >80% power with α < 0.05. To account for incomplete data and other factors, the sample size was increased to 650. Thus, the total sample size was 650 mother-child pairs.

MS Excel (Microsoft Corporation, Redmond, Washington, United States) spreadsheet was used to enter the data and checked for errors and discrepancies. Data analysis was done using Windows-based MedCalc Statistical Software version 19.0.1 (MedCalc Software Ltd., Ostend, Belgium). Demographic data were analyzed using the chi-squared test. The independent samples t-test was used to compare the ECOHIS scores of children with mothers having high dental anxiety and those having low dental anxiety (based on CDAS scores). The association between CDAS scores and ECOHIS scores of children was analyzed using Pearson’s correlation coefficient. ANOVA was used for intergroup comparison of CDAS scores and ECOHIS scores based on children’s age, sex, maternal age, maternal education, and SES. All testing was done at a significance of 0.05.

## Results

The data for 649 mother-child pairs were analyzed after one of the mothers withdrew from the study midway.

Table 1 summarizes the demographic characteristics of the study population. Boys constituted 52.08% (n=338) of the study population. A majority of the mothers, 63% (n=408), belonged to the age range of 30-40 years, and most of them, 53% (n=342), had a graduate or postgraduate qualification.

**Table 1:** Demographic data of the study population N=649; #: chi-squared test; *: statistically significant

Variables	Category	n (%)	x^2#^	p
Age of children	3 years	23 (3.54)	8.26	0.04*
	4 years	152 (23.42)		
	5 years	248 (38.21)		
	6 years	226 (34.82)		
Sex of children	Boys	338 (52.08)	1.11	0.29
	Girls	311 (47.92)		
Mother’s age	20-30 years	206 (31.74)	322.30	<0.001*
	30-40 years	408 (62.86)		
	>40 years	35 (5.39)		
Mother’s education	Primary School	7 (1.07)	694.17	<0.001*
	Middle School	15 (2.31)		
	High School	97 (14.94)		
	Intermediate	116 (17.87)		
	Degree	72 (11.09)		
	Graduate/Post Graduate	342 (52.69)		

Table 2 compares the CDAS and ECOHIS (CIS, FIS, and total ECOHIS) scores based on the age of the children. There was no statistically significant difference in mean maternal dental anxiety scores between children aged three and six years (f=0.816, p > 0.05). The mean ECOHIS scores were highest in children aged six years and lowest in children aged three years, the difference being statistically significant (f=11.300, p < 0.0001).

**Table 2:** Intergroup comparison of CDA and ECOHIS (CIS, FIS, and total ECOHIS) scores w.r.t. age of children CDA: Corah’s Dental Anxiety; ECOHIS: Early Childhood Oral Health Impact Scale; CIS: Child Impact Section; FIS: Family Impact Section N=649; #: ANOVA; *: statistically significant

Age (n)	CDA (mean±SD) (95% CI)	ECOHIS (child domain) (mean±SD) (95% CI)	ECOHIS (family domain) (mean±SD) (95% CI)	ECOHIS (Total) (mean±SD) (95% CI)
3 (23)	10.48 ± 2.921 (9.22,11.74)	9.65 ± 7.15 (6.56,12.75)	4.61 ± 3.56 (3.07,6.15)	14.26 ± 9.923 (9.97,18.55)
4 (152)	9.92 ± 3.327 (9.39,10.45)	14.93 ± 8.272 (13.60,16.25)	14.47 ± 10.62 (12.76,16.17)	29.39 ± 18.415 (26.44,32.35)
5 (248)	10.42 ± 3.000 (10.04,10.79)	16.74 ± 7.804 (15.76,17.71)	16.13 ± 10.80 (14.78,17.48)	32.87 ± 17.946 (30.63,35.12)
6 (226)	10.22 ± 3.309 (9.78,10.65)	17.42 ± 7.981 (16.37,18.47)	17.86 ± 10.68 (16.46,19.26)	35.28 ± 18.093 (32.91,37.65)
^f^^ #^	0.816	8.603	12.353	11.300
p	0.485	<0.0001*	<0.0001*	<0.0001*

Table 3 compares the CDAS and ECOHIS (CIS, FIS, and total ECOHIS) scores based on the sex of the children. The maternal dental anxiety scores in boys were higher than in girls; however, the difference was not significant (t=0.539, p > 0.05). The ECOHIS scores were higher in girls compared to boys; however, the difference was not statistically significant (t=1.250, p > 0.05).

**Table 3:** Intergroup comparison of CDA and ECOHIS (CIS, FIS, and total ECOHIS) scores w.r.t. sex of children CDA: Corah’s Dental Anxiety; ECOHIS: Early Childhood Oral Health Impact Scale; CIS: Child Impact Section; FIS: Family Impact Section N=649; ##: student t-test; *: statistically significant

Sex (n)	CDA (mean±SD) (95% CI)	ECOHIS (child domain) (mean±SD) (95% CI)	ECOHIS (family domain) (mean±SD) (95% CI)	ECOHIS (Total) (mean±SD) (95% CI)
Boys (338)	10.30 ± 3.31 (9.94,10.65)	15.94 ± 7.75 (15.11,16.76)	15.43 ± 10.32 (14.33,16.53)	31.38 ± 17.46 (29.52,33.24)
Girls (311)	10.16 ± 3.04 (9.82,10.49)	16.69 ± 8.45 (15.75,17.62)	16.49 ± 11.34 (15.23,17.75)	33.17 ± 19.19 (31.03,35.30)
t^##^	0.539	1.170	1.239	1.250
p	0.590	0.242	0.216	0.212

A moderate positive correlation was observed between CDA scores and ECOHIS scores in the child domain (r=0.476; p < 0.001), family domain (r=0.38; p < 0.001), and total (r=0.422; p < 0.001), which was statistically significant across all three domains (Table 4).

**Table 4:** Association of CDA with OHRQoL CDA: Corah’s Dental Anxiety; OHRQoL: oral health-related quality of life; ECOHIS: Early Childhood Oral Health Impact Scale N=649; #: Pearson’s correlation coefficient; *: statistically significant

		CDA (Total Score)	ECOHIS (child domain)	ECOHIS (family domain)	ECOHIS (Total)
CDA	r^#^	-	0.476	0.38	0.422
	p	-	<0.0001*	<0.0001*	<0.0001*
ECOHIS (child domain)	r^#^	0.476	-	0.859	0.955
	p	<0.0001*	-	<0.0001*	<0.0001*
ECOHIS (family domain)	r^#^	0.36	0.859	-	0.97
	p	<0.0001*	<0.0001*	-	<0.0001*
ECOHIS (Total)	r^#^	0.422	0.955	0.97	-
	p	<0.0001*	<0.0001*	<0.0001*	-

The mean ECOHIS scores in children with high maternal dental anxiety were higher than in those with low maternal dental anxiety, the difference being statistically significant (t=10.420, p < 0.0001; Table 5).

**Table 5:** Intergroup comparison of ECOHIS scores between mothers with low dental anxiety and high dental anxiety ECOHIS: Early Childhood Oral Health Impact Scale N=649; #: student t-test; *: statistically significant

Maternal Dental Anxiety (n)	ECOHIS (child domain) (mean±SD) (95% CI)	ECOHIS (family domain) (mean±SD) (95% CI)	ECOHIS (Total) (mean±SD) (95% CI)
Low anxiety (401)	13.71 ± 6.85 (13.04,14.38)	13.07 ± 8.96 (12.19,13.94)	26.78 ± 15.04 (25.30,28.25)
High anxiety (248)	20.49 ± 8.21 (19.46,21.51)	20.57 ± 11.94 (19.08,22.05)	41.06 ± 19.68 (38.61,43.50)
t^#^	11.337	9.103	10.420
p	<0.0001*	<0.0001*	<0.0001*

A statistically significant difference in maternal dental anxiety scores and ECOHIS scores (all domains) was seen across maternal age, maternal education, and SES (Table 6).

**Table 6:** Intergroup comparison of CDA and ECOHIS (CIS, FIS, and total ECOHIS) scores w.r.t. maternal age, maternal education, and SES CDA: Corah’s Dental Anxiety; OHRQoL: oral health-related quality of life; ECOHIS: Early Childhood Oral Health Impact Scale; CIS: Child Impact Section; FIS: Family Impact Section; SES: socioeconomic status N=649; #: ANOVA; *: statistically significant

Maternal Age (n)	CDA (mean±SD) (95% CI)	ECOHIS (child domain) (mean±SD) (95% CI)	ECOHIS (family domain) (mean±SD) (95% CI)	ECOHIS (Total) (mean±SD) (95% CI)
>40 yrs (35)	8.54 ± 2.822 (7.57,9.51)	11.29 ± 7.078 (8.85, 13.72)	11.17 ± 8.989 (8.08, 14.26)	22.46 ± 15.683 (17.07,27.84)
30-40 yrs (408)	10.11 ± 3.149 (9.81,10.42)	15.92 ± 7.935 (15.15, 16.69)	16.12 ± 10.478 (15.10,17.14)	32.04 ± 17.920 (30.30, 33.79)
20-30 yrs (206)	10.76 ± 3.202 (10.32,11.20)	17.91 ± 8.165 (16.79, 19.03)	16.38 ± 11.623 (14.78, 17.98)	34.29 ± 19.018 (31.67,36.90)
f^ #^	8.246	11.595	3.651	6.406
p	<0.0001*	<0.0001*	0.027*	0.002*
Maternal Education				
Primary school	11.50 ± 2.27	13.75 ± 3.92	4.88 ± 1.89	18.63 ± 3.89
Middle school	10.09 ± 3.91	14.45 ± 6.58	7.27 ± 5.62	21.73 ± 10.35
High school	10.89 ± 3.67	14.36 ± 8.51	10.76 ± 11.23	25.11 ± 18.91
Intermediate/ Diploma	10.64 ± 2.94	20.14 ± 7.24	22.30 ± 8.93	42.44 ± 16.00
Graduate/Post-graduate	10.06 ± 3.09	15.64 ± 8.16	15.21 ± 10.61	30.85 ± 18.11
Professional degree	9.36 ± 3.04	16.44 ± 7.21	18.79 ± 8.94	35.23 ± 16.10
f^ #^	2.762	7.476	19.324	13.85
p	0.018*	<0.0001*	<0.0001*	<0.0001*
SES				
Upper lower class	9.70 ± 3.62	13.78 ± 5.27	6.96 ± 3.89	20.74 ± 7.42
Lower middle class	10.88 ± 3.26	12.54 ± 7.38	6.33 ± 6.55	18.87 ± 12.62
Upper middle class	10.46 ± 3.13	17.64 ± 8.34	17.89 ± 11.08	35.53 ± 18.91
Upper class	9.59 ± 3.10	15.58 ± 7.56	17.27 ± 9.67	32.85 ± 17.06
f^#^	4.502	10.61	35.481	22.95
p	0.004*	<0.0001*	<0.0001*	<0.0001*

## Discussion

The most important caregiver for a child is usually the mother, and so, in situations such as fear-provoking, she may influence her child’s development of anxious or coping responses [16]. Children may learn from their mothers via modeling, information, reinforcement, or even more subtle forms of communication [16]. Because mothers usually spend more time with the child at an early age, mothers with high anxiety levels have often been shown to exert a negative influence on their children’s behavior in the dental office [10,17,18]. Dental anxiety creates a vicious cycle of avoidance behavior, resulting in fewer dental visits and more appointment cancellations [9], which may have a negative impact on oral health.

The prevalence of maternal dental anxiety in the study population was 38.21%. The age of the children had a significant impact on OHRQoL. As age increased, ECOHIS scores increased, indicating that older children had poorer OHRQoL, which is in agreement with previous reports by Pakkhesal (2021) [19]. Sex of the children had no impact on maternal dental anxiety or OHRQoL, indicating that the sex of the child was not a determinant of maternal dental anxiety or OHRQoL. Previous study findings by Soltani et al. also indicated a non-significant relationship between sex and OHRQoL [20]. Younger mothers were more anxious. Mohammed et al. (2024) reported that the anxiety levels of younger age groups were significantly higher than those of older age groups [21].

We found a moderate positive correlation between CDAS and ECOHIS scores across all domains. As CDAS scores increased, ECOHIS scores increased. Higher ECOHIS scores are indicative of a poorer OHRQoL. Thus, maternal dental anxiety had a negative impact on the OHRQoL of children. We also found a significant difference in the ECOHIS scores across all domains in mothers who were less anxious compared to highly anxious mothers. Goettems et al. concluded that maternal dental anxiety had a negative effect on the perception of the impact of the oral health problems of the child on the family, thus affecting the parent distress domain [10]. A significant impact of maternal dental anxiety and the child’s dental caries experience on the child’s OHRQoL has been reported by Esa and co-workers [22].

Maternal age had a significant impact on the maternal dental anxiety and OHRQoL of children. Mothers older than 40 years had less maternal dental anxiety, and their children had a better OHRQoL. Mean CDA scores reduced with increasing age. This suggests that as the age of the mother increased, maternal dental anxiety decreased, and OHRQoL in children improved. Our study results agree with previous studies that have reported similar findings [23,24]. Locker et al. suggested that the age-dependent mental degenerative factors, like extinction or habituation, and adaptive surrender or inevitability [25]. Mohammed et al. reported that the overall prevalence of dental anxiety was high in the 25-35-year age group and low in the 55-65-year age group, which is in accordance with our study [21].

Children of mothers older than 40 years of age had a better OHRQoL. Yang has reported that younger parents feel less secure or have less experience in caring for their children and are more likely to feel guilty because of their children’s dental problems [23], leading to an increase in ECOHIS scores. Conversely, Abanto et al. and Martins-Júnior et al. found that there is no significant association between parents’ age and their children’s OHRQoL [26,27].

We found out that middle-class mothers were more anxious than upper- or lower-class mothers. However, other studies have concluded that poor SES created more dental anxiety [28]. This could be partly attributed to the cost of the dental treatment, which is deemed to be a more expensive treatment modality.

Maternal education had a significant impact on both maternal dental anxiety and the OHRQoL of children. Less educated mothers had higher dental anxiety. Consistent with our study results, Fayad et al. also found that patients with lower levels of education had higher anxiety scores [29]. In contrast, Alansaari et al. suggested that as educational level increases, so do dental anxiety scores [30].

In our study, OHRQoL was poorer in children of mothers with intermediate education. Yang reported significantly lower scores in mothers with higher education levels compared to those with an education level of college or less [24].

Strengths and limitations

The strength of this study lies in the use of validated tools (CDAS and ECOHIS), which have been widely used across different populations. Additionally, the impact of covariables like child age, maternal age, mother’s education, and SES on maternal dental anxiety and OHRQoL of children was also assessed.

The limitation of the study was that this research could only explore the association between maternal dental anxiety and the child’s OHRQoL, in view of it following a cross-sectional study design. Though the temporality of maternal dental anxiety is logical, causality could not be established. Moreover, the use of the ECOHIS relies on self-reported parental perceptions, which may not accurately reflect the child’s actual OHRQoL, thus introducing an element of bias. In view of the above, the results of this study have limited generalizability.

## Conclusions

Overall, maternal dental anxiety had a negative impact on the OHRQoL of children. Children whose mothers had higher dental anxiety had a poorer OHRQoL. Maternal age and maternal education had a significant impact on maternal dental anxiety and OHRQoL of children; younger and less educated mothers were more anxious, and their children had a poor OHRQoL. Additionally, older children had a poor OHRQoL.

Based on the conclusions of our study, we would recommend a multicentric study, including an analysis of other confounders, to further substantiate the claims made. Since maternal dental anxiety can negatively impact the OHRQoL of children, adequate and positive communication between the dentist and the mother, using techniques like pre-appointment counseling, could therefore help in addressing this factor and would contribute to better utilization of dental services and improving the OHRQoL of children.
